# Incidence of postoperative opioid-induced respiratory depression episodes in patients on room air or supplemental oxygen: a post-hoc analysis of the PRODIGY trial

**DOI:** 10.1186/s12871-023-02291-x

**Published:** 2023-10-04

**Authors:** Anthony G. Doufas, Mariana L. Laporta, C. Noelle Driver, Fabio Di Piazza, Marco Scardapane, Sergio D. Bergese, Richard D. Urman, Ashish K. Khanna, Toby N. Weingarten, The Prediction of Opioid-induced respiratory Depression In patients monitored by capnoGraphY (PRODIGY) Group Investigators, Carla R. Jungquist, Hiroshi Morimatsu, Shoichi Uezono, Simon Lee, Lian Kah Ti, Robert McIntyre, Carlos Tornero, Albert Dahan, Leif Saager, Maria Wittmann, Dennis Auckley, Luca Brazzi, Morgan Le Guen, Roy Soto, Frank Schramm, Wolfgang Buhre, Frank J. Overdyk

**Affiliations:** 1 Department of Anesthesiology, Perioperative and Pain Medicine, Center for Sleep and Circadian Sciences, Stanford University School of Medicine, 300 Pasteur Drive, H3580, Stanford, San Francisco, CA 94305-5640 USA; 2 Department of Anesthesiology and Perioperative Medicine, Mayo Clinic, Rochester, MN USA; 3 Medtronic Core Clinical Solutions, Global Clinical Data Solutions, Rome, Italy; 4 Department of Anesthesiology and Neurological Surgery, Stony Brook University School of Medicine, Stony Brook, New York, USA; 5 Department of Anesthesiology, The Ohio State University and Wexner Medical Center, Columbus, OH USA; 6 Section On Critical Care Medicine, Department of Anesthesiology, Wake Forest Center for Biomedical Informatics, Perioperative Outcomes and Informatics Collaborative (POIC), Wake Forest University School of Medicine, Winston-Salem, North Carolina USA; 7 Outcomes Research Consortium, Cleveland, OH USA

**Keywords:** Respiratory depression, Apnea, Supplemental oxygen, Room air, Capnography, Pulse oximetry, Continuous monitoring, Spot check monitoring

## Abstract

**Background:**

Supplemental oxygen (SO) potentiates opioid-induced respiratory depression (OIRD) in experiments on healthy volunteers. Our objective was to examine the relationship between SO and OIRD in patients on surgical units.

**Methods:**

This post-hoc analysis utilized a portion of the observational PRediction of Opioid-induced respiratory Depression In patients monitored by capnoGraphY (PRODIGY) trial dataset (202 patients, two trial sites), which involved blinded continuous pulse oximetry and capnography monitoring of postsurgical patients on surgical units. OIRD incidence was determined for patients receiving room air (RA), intermittent SO, or continuous SO. Generalized estimating equation (GEE) models, with a Poisson distribution, a log-link function and time of exposure as offset, were used to compare the incidence of OIRD when patients were receiving SO vs RA.

**Results:**

Within the analysis cohort, 74 patients were always on RA, 88 on intermittent and 40 on continuous SO. Compared with when on RA, when receiving SO patients had a higher risk for all OIRD episodes (incidence rate ratio [IRR] 2.7, 95% confidence interval [CI] 1.4–5.1), apnea episodes (IRR 2.8, 95% CI 1.5–5.2), and bradypnea episodes (IRR 3.0, 95% CI 1.2–7.9). Patients with high or intermediate PRODIGY scores had higher IRRs of OIRD episodes when receiving SO, compared with RA (IRR 4.5, 95% CI 2.2–9.6 and IRR 2.3, 95% CI 1.1–4.9, for high and intermediate scores, respectively).

**Conclusions:**

Despite oxygen desaturation events not differing between SO and RA, SO may clinically promote OIRD. Clinicians should be aware that postoperative patients receiving SO therapy remain at increased risk for apnea and bradypnea.

**Trial registration:**

Clinicaltrials.gov: NCT02811302, registered June 23, 2016.

**Supplementary Information:**

The online version contains supplementary material available at 10.1186/s12871-023-02291-x.

## Background

Fatal or seriously debilitating consequences of postoperative opioid-induced respiratory depression (OIRD) [1] are rare, however, a large fraction of surgical patients experience prolonged and persistent hypoxemia [2]. Importantly, these patients might be at risk for major postoperative pulmonary complications [3], despite the use of supplemental oxygen (SO) [1, 2, 4].

Oxygen (O_2_) is a drug with powerful actions on several aspects of ventilatory control [5]. Through its effect on the peripheral chemoreceptors at the carotid body, O_2_ directly suppresses ventilatory drive [6], and decreases the sensitivity of central chemoreceptors to carbon dioxide [7]. In addition, at high inspired fractions, O_2_ reduces cerebral blood flow and may thus increase the apparent potency of opioids [8], by raising their concentration at the effect site [9]. If applicable, these effects of SO would tend to enhance hypoventilation to a clinical setting. On the other hand, although SO is necessary to increase oxyhemoglobin saturation in postoperative patients [10], it doesn’t seem to affect the number of episodic desaturations [11]. This indicates that when continuous oximetry is the only available monitor, an increase in oxygenation by SO might conceal and potentially delay the detection of severe respiratory depression [12, 13], which, thus, might quickly precipitate to frank apnea and hypoxemia. Expectedly, the situation may become even more critical when during SO, oxygenation is not monitored continuously, but intermittently assessed by nurses, since spot-checks of oxygenation might miss up to 90% of clinical hypoxemia episodes [2].

The purpose of this post-hoc analysis of PRODIGY (PRediction of Opioid-induced respiratory Depression In patients monitored by capnoGraphY) observational trial was to examine the association between oxygen supplementation and the incidence and pattern of OIRD in patients admitted to surgical hospital wards. This is an environment where standard of care monitoring usually consists of spot-checks of oxygenation, respiratory rate, and hemodynamic parameters.

## Methods

The PRODIGY trial (registered prior to patient enrollment at clinicaltrials.gov: NCT02811302, principal investigator: Frank J. Overdyk, https://clinicaltrials.gov/ct2/show/NCT02811302, date of registration: June 23, 2016) was a prospective, observational trial conducted between April 2017 and May 2018 in the United States, Europe, and Asia. The trial was approved by each institutional review board or ethics committee, and all patients provided written informed consent before enrollment. This analysis included data from 2 United States trial sites (Brigham & Women’s Hospital, Boston, MA and The Ohio State University Wexner Medical Center, Columbus, OH). This manuscript adheres to the applicable Strengthening the Reporting of OBservational studies in Epidemiology (STROBE) guidelines.

### Initial trial design

The objective of the original PRODIGY trial was to develop a risk prediction tool to identify patients at risk of OIRD on the medical/surgical unit [14]. Eligible patients included adult patients who were expected to receive parenteral opioids in the medical/surgical unit after a procedure or surgery. Exclusion criteria were: 1) Patients whose hospital stay was anticipated to be < 24 h; 2) Patients who received intrathecal opioids; 3) Patients with American Society of Anesthesiology (ASA) physical status V or higher; 4) Patients with an active Do Not Resuscitate status; 5) Patients who were expected to be ventilated or intubated; 6) Patients unwilling or unable to comply with monitoring procedures; 7) Patients who were part of a vulnerable population; and 8) Patients who were participating in a confounding clinical trial [14]. After arrival on the medical/surgical unit, enrolled patients underwent blinded, continuous pulse oximetry and capnography monitoring (Capnostream™ 20p or 35 portable respiratory monitor, Medtronic, Boulder, CO). The median length of continuous monitoring was 24h (interquartile range 17-26h), during which standard of care spot-check monitoring was performed per site protocol [1]. Supplemental oxygen (SO) was provided based on local site practices.

### Definition criteria for OIRD

An OIRD episode was pre-defined as: respiratory rate ≤ 5 breaths/min (bpm), oxygen saturation ≤ 85%, or end-tidal carbon dioxide ≤ 15 or ≥ 60 mm Hg for ≥ 3 min; apnea episode lasting > 30 s; or any respiratory opioid-related adverse event [1]. Potential OIRD episodes were identified by monitor algorithms and independently assessed by a panel of capnography experts, blinded to patient medical history, including SO use, to categorize patients as having either ≥ 1 OIRD episode or no OIRD [1, 15]. Patients with > 1 potential OIRD episode underwent OIRD adjudication only until the first OIRD episode was confirmed. This adjudicated data was then used to develop the PRODIGY risk prediction tool, which assesses 5 patient characteristics (age > 60 in decades, male sex, sleep-disordered breathing, opioid naivety, and chronic heart failure) to determine whether a patient has low, intermediate, or high risk for OIRD [1].

### Objectives

The objective of this post-hoc analysis was to determine whether patients on room air (RA) experience more OIRD episodes, compared with when receiving intermittent or continuous SO. Secondary objectives were to assess the type of OIRD episodes occurring in patients receiving intermittent or continuous SO or on RA, evaluate the association between OIRD episodes during intermittent or continuous SO or on RA as a function of PRODIGY score and finally, examine the frequency of OIRD episodes as a function of the time after the end of surgery.

### OIRD episode assessment

This analysis included data from surgical patients enrolled at 2 United States trial sites and utilized the OIRD episode dataset from a previous post-hoc analysis, in which all potential OIRD episodes were adjudicated, including each potential OIRD episode in patients who had > 1 OIRD episode [15]. The incidence of OIRD episode was compared among 3 groups of patients: those always on RA, those with intermittent SO use, and those always receiving SO during continuous monitoring. The subgroup analysis on data from 88 patients with intermittent SO use, was performed to mitigate potential confounding factors that may exist between patients who always received SO and those breathing RA during continuous monitoring.

### Statistical analysis

This was a post-hoc analysis, and therefore the sample size was determined by data availability. Patients without complete SO records during the continuous monitoring period were excluded from the analysis. Descriptive and baseline characteristics were reported using mean and standard deviation (SD) or frequencies and percentages for continuous and categorical variables, respectively. The incidence of OIRD episodes was modeled using generalized estimating equation (GEE) models, with a Poisson distribution, a log-link function and time of exposure as offset. Estimated differences between groups were reported as incidence rate ratios (IRR), along with their 95% CI. Subtypes of OIRD, including low respiratory rate, low arterial blood oxygen saturation (SpO_2_), and apnea episodes, were also assessed separately using GEE models. A GEE model was also used to evaluate the incidence of OIRD episodes stratifying the analysis by patient PRODIGY score. All tests were two-sided and p-values < 0.05 were considered statistically significant. Analyses were performed using SAS® Version 9.4 (SAS Institute Inc., Cary, NC).

## Results

### Participants

**Fig. 1:**
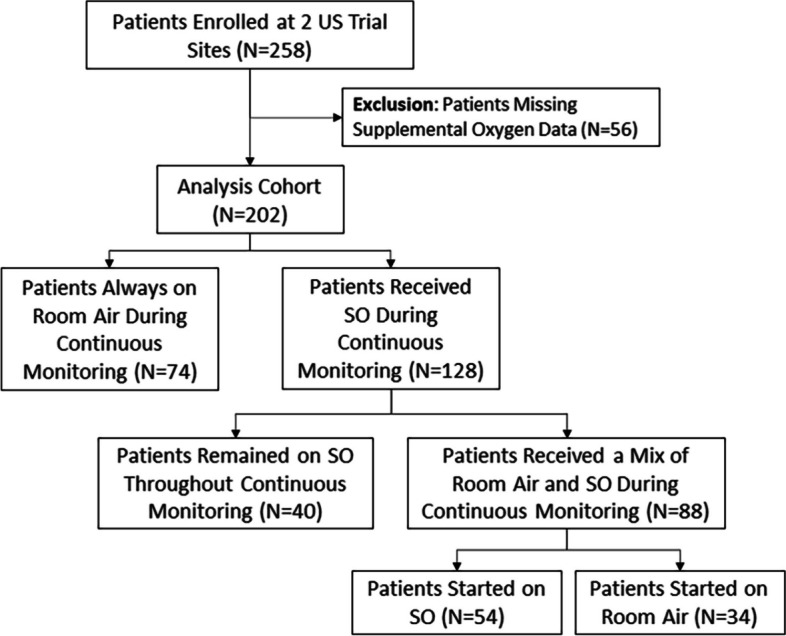
Flow chart of PRODIGY patients from 2 trial sites receiving supplemental oxygen during continuous monitoring. Abbreviations: PRODIGY = PRediction of Opioid-induced respiratory Depression In patients monitored by capnoGraphY; SO = Supplemental oxygen

Among 258 patients enrolled at 2 United States trial sites in this post-hoc analysis, 56 patients had incomplete SO data records and were excluded from the analysis (Fig. 1). Of the 202 patients in the analysis cohort, 74 remained on RA throughout continuous monitoring on the surgical unit. Forty patients received SO for the entirety of the continuous monitoring period, and 88 patients received intermittent supplemental oxygen during continuous monitoring (Fig. 1). Within these 88 patients, 54 patients started continuous monitoring with SO and discontinued SO later during the monitoring period, and 34 patients started continuous monitoring on RA and received SO later during the monitoring period.

### Demographics and medical history

Among 74, 88, and 40 patients on RA, intermittent, or continuous SO during continuous monitoring on the surgical unit, the average age was 49 ± 16, 53 ± 13, and 56 ± 14 years, respectively (*p* = 0.053), (Table 1). Most patients were ASA class III, independent of SO use. Patient PRODIGY score, which was retrospectively calculated and represents the risk of OIRD, was high (Additional File 1) for 19% of patients always on RA, 26% of patients intermittently receiving SO, and 33% of patients always on SO. Between 81 and 85% of patients were opioid naïve, and > 98% received multiple opioids or concurrent central nervous system (CNS)/sedating medication, regardless of SO use. Postoperative morphine milligram equivalents did not differ significantly among groups (Table 1).

**Table 1:** Characteristics of patients breathing room air, intermittently receiving SO, or always receiving SO

Patient Characteristics	Always Room Air (*n* = 74)	Intermittent SO (*n* = 88)	Always SO (*n* = 40)	*p*-value
**Age (yrs) (**Mean ± SD)	49.4 ± 15.5	52.6 ± 12.9	56.0 ± 13.9	0.053
**Body Mass Index (kg/m**^**2**^**) (**Mean ± SD)	36.6 ± 12.3	36.2 ± 11.1	34.5 ± 12.6	0.440
**Current Smoker**	8.1% (6/74)	8.0% (7/88)	5.0% (2/40)	0.807
**Oxygen Use at Home**	0.0% (0/74)	1.1% (1/88)	2.5% (1/40)	1.0
**ASA Physical Status**				
ASA I	0.0% (0/74)	0.0% (0/88)	0.0% (0/40)	0.603
ASA II	28.4% (21/74)	22.7% (20/88)	30.0% (12/40)	
ASA III	71.6% (53/74)	76.1% (67/88)	67.5% (27/40)	
ASA IV	0.0% (0/74)	1.1% (1/88)	2.5% (1/40)	
**PRODIGY Score**				
Low (< 8)	53.4% (39/73)	43.2% (38/88)	27.5% (11/40)	0.123
Intermediate (8–14)	27.4% (20/73)	30.7% (27/88)	40.0% (16/40)	
High (≥ 15)	19.2% (14/73)	26.1% (23/88)	32.5% (13/40)	
**SO Flow Rate (L/min) (**Mean ± SD)	NA	2.1 ± 0.7	1.9 ± 0.7	0.538
**Surgical Demographics**				
High-Risk Surgery^a^	6.8% (5/74)	11.4% (10/88)	17.5% (7/40)	0.210
Open Surgery	12.2% (9/74)	17.0% (15/88)	10.0% (4/40)	0.490
**Opioid Demographics**				
Opioid Naive	81.1% (60/74)	84.1% (74/88)	85.0% (34/40)	0.827
Multiple Opioids or concurrent CNS/Sedating Medication	100.0% (74/74)	97.7% (86/88)	100.0% (40/40)	0.270
Opioid Morphine Milligram Equivalents (post-surgery) (Median, IQR)	26.9 (7.6- 90.0)	27.1 (15.3- 60.2)	21.5 (12.4- 76.6)	0.798
**Medical History**				
**Cardiac Disorders**				
Aortic Aneurysm	0.0% (0/74)	2.3% (2/88)	0.0% (0/40)	0.679
Aortic Valve Disease	0.0% (0/74)	3.4% (3/88)	0.0% (0/40)	0.309
Chronic Heart Failure	1.4% (1/73)	2.3% (2/88)	0.0% (0/40)	1.000
Coronary Artery Disease	4.1% (3/73)	1.1% (1/88)	5.0% (2/40)	0.353
Hypertension	43.2% (32/74)	56.8% (50/88)	47.5% (19/40)	0.214
Mitral Valve Disease	1.4% (1/74)	1.1% (1/88)	0.0% (0/40)	1.000
Myocardial Infarction	0.0% (0/73)	2.3% (2/88)	0.0% (0/40)	0.680
**Sepsis**	1.4% (1/74)	1.1% (1/88)	0.0% (0/40)	1.000
**Metabolism and Nutrition Disorders**				
Diabetes—Type I	1.4% (1/74)	3.4% (3/88)	0.0% (0/40)	0.537
Diabetes—Type II	21.6% (16/74)	14.8% (13/88)	15.0% (6/40)	0.471
**Kidney Failure**	1.4% (1/74)	2.3% (2/88)	2.5% (1/40)	1.000
**Respiratory Disorders**				
Acute Bronchitis	0.0% (0/74)	1.1% (1/88)	5.0% (2/40)	0.149
Asthma	23.0% (17/74)	21.6% (19/88)	7.5% (3/40)	0.105
Chronic Bronchitis	0.0% (0/74)	1.1% (1/88)	0.0% (0/40)	1.000
Chronic Obstructive Pulmonary	5.4% (4/74)	4.5% (4/88)	5.0% (2/40)	1.000
Pneumonia	0.0% (0/74)	2.3% (2/88)	5.0% (2/40)	0.191
**Sleep Disordered Breathing**^**b**^	14.9% (11/74)	20.5% (18/88)	25.0% (10/40)	0.398
**Vascular Disorders**				
Cerebral Aneurysm	2.7% (2/74)	4.5% (4/88)	0.0% (0/40)	0.427
Peripheral Vascular Disease	1.4% (1/74)	2.3% (2/88)	2.5% (1/40)	1.000
Stroke	4.1% (3/73)	3.4% (3/88)	2.5% (1/40)	1.000
Transient Ischemic Attack	2.7% (2/73)	1.1% (1/88)	2.5% (1/40)	0.678

*Abbreviations SO* Supplemental oxygen, *SD* Standard deviation, *ASA* American Society of Anesthesiologists, *PRODIGY* PRediction of Opioid-induced respiratory Depression In patients monitored by capnography, *CNS* Central nervous system, *IQR* Interquartile range, *NA* Not applicable, *L* Liters, min, Minute, *ESC/ESA* European Society of Cardiology/European Society of Anaesthesiology

^a^High-risk surgery was defined using the revised ESC/ESA guidelines on non-cardiac surgery

^b^Known or suspected sleep-disordered breathing included medical history of obstructive sleep apnea, the use of continuous positive airway pressure (CPAP), or confirmation of the Snoring, Tiredness, Observed apnea, blood Pressure (STOP) questions in the Snoring, Tiredness, Observed apnea, blood Pressure, Body mass index, Age, Neck circumference and Gender (STOP-BANG) questionnaire

### Respiratory depression occurrence

At least one OIRD episode occurred in 54% (*N* = 40/74), 60% (*N* = 53/88), and 63% (*N* = 25/40) of patients always on RA, intermittently receiving SO, and always on SO, respectively (Table 2). Compared with patients always on RA or receiving intermittent SO, the number of OIRD episodes per patient per monitored hour was significantly higher in patients always on SO (*p* = 0.017). Patients always on SO also had a significantly higher number of apnea episodes per hour, compared to patients always on RA or receiving intermittent SO (*p* = 0.005). The occurrence of respiratory rate and SpO_2_ episodes did not differ between the 3 groups (Table 2).

**Table 2:** Respiratory depression occurrence when patients were breathing room air, intermittently receiving SO, or always receiving SO

Respiratory Depression Episode	Always Room Air (*n* = 74)	Intermittent SO (*n* = 88)	Always SO (*n* = 40)	*p*-value
** ≥ 1 OIRD Episode**				
Percent of patients with ≥ 1 OIRD episode (n/N)	54.1% (40/74)	60.2% (53/88)	62.5% (25/40)	0.615
Number of OIRD episodes per patient (Median, IQR)	4.5 (2.0- 9.5)	6.0 (2.0- 18.0)	8.0 (2.0- 20.0)	0.420
Number of OIRD episodes per patient per monitored hour (Median, IQR)	0.3 (0.1- 0.4)	0.3 (0.1- 0.6)	0.9 (0.2- 1.3)	0.017
**Respiratory Depression Episode Subtype**				
** ≥ 1 Apnea Episode**				
Percent of patients with ≥ 1 apnea episode	48.6% (36/74)	56.8% (50/88)	60.0% (24/40)	0.427
Number of apnea episodes per patient (Median, IQR)	4.0 (2.0- 9.0)	5.0 (2.0- 17.0)	8.0 (2.5- 18.5)	0.238
Number of apnea episodes per patient per monitored hour (Median, IQR)	0.2 (0.1- 0.4)	0.3 (0.1- 0.6)	0.7 (0.3- 1.3)	0.005
** ≥ 1 Respiratory Rate Episode**				
Percent of patients with ≥ 1 respiratory rate episodes	13.5% (10/74)	19.3% (17/88)	12.5% (5/40)	0.488
Number of respiratory rate episodes per patient (Median, IQR)	1.0 (1.0- 1.0)	1.0 (1.0- 2.0)	3.0 (2.0- 3.0)	0.096
Number of RR episodes per patient per monitored hour (Median, IQR)	0.1 (0.0- 0.1)	0.1 (0.0- 0.1)	0.1 (0.1- 0.1)	0.108
** ≥ 1 SpO**_**2**_** Episode**				
Percent of patients with ≥ 1 SpO_2_ episodes	9.5% (7/74)	9.1% (8/88)	12.5% (5/40)	0.825
Number of SpO_2_ episodes per patient (Median, IQR)	3.0 (1.0- 9.0)	1.0 (1.0- 2.5)	1.0 (1.0- 3.0)	0.157
Number of SpO_2_ episodes per patient per monitored hour (Median, IQR)	0.1 (0.1- 0.5)	0.1 (0.0- 0.1)	0.0 (0.0- 0.1)	0.241

*Abbreviations* SO, Supplemental oxygen, *OIRD* Opioid-induced respiratory depression, *IQR* Interquartile range

**Fig. 2:**
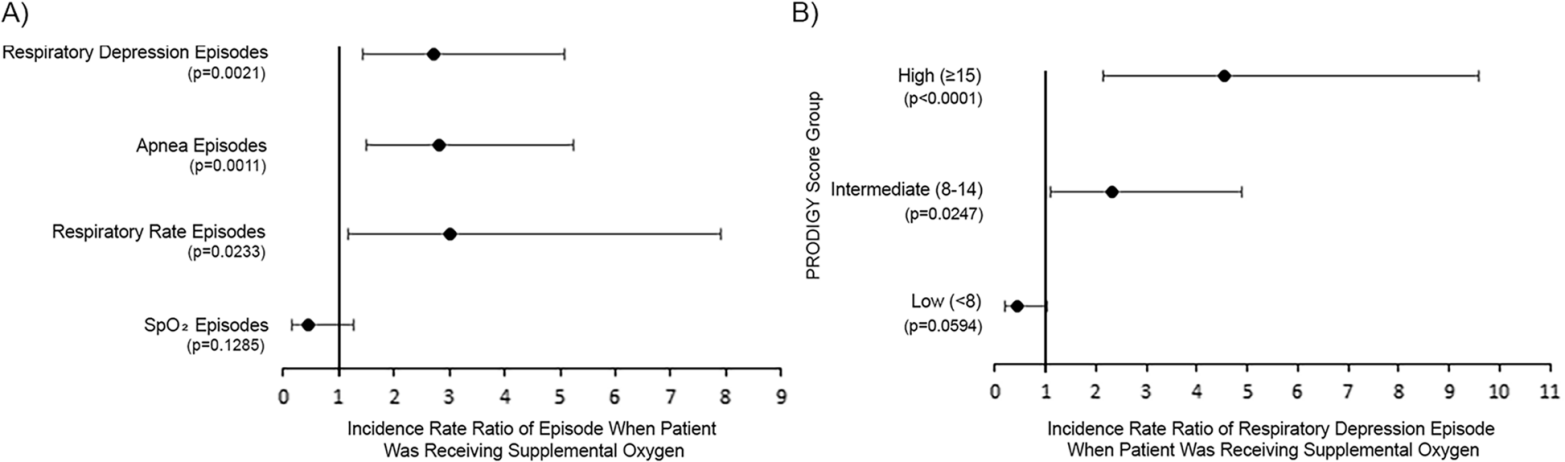
Generalized estimating equation model for **A** the incidence rate ratio of respiratory depression episodes in patients during times of supplemental oxygen, compared with breathing room air, and **B** the incidence rate ratio of respiratory depression episodes in patients on supplemental oxygen, compared with breathing room air, as a function of PRODIGY score. Analysis includes full dataset (*N* = 202)

To account for internal correlation within patients between the time on RA and SO, a GEE model was used. Based on this analysis, the incidence rate ratio for OIRD episodes was 2.7 (95% CI 1.4–5.1, *p* = 0.0021), and the incidence rate ratios for apnea episodes or respiratory rate episodes were 2.8 (95% CI 1.5–5.2, *p* = 0.0011) and 3.0 (95% CI 1.2–7.9, *p* = 0.0233), respectively, when receiving SO (Fig. 2A). These results were confirmed by a subgroup analysis using the 88 patients who received intermittent SO, to control for potential differences between patients always on SO and patients always on RA (Additional File 2). The incidence rate ratio for OIRD episodes was also significantly higher among patients with a high or intermediate PRODIGY score when they were receiving SO (IRR 4.5, 95% CI 2.2–9.6, p < 0.0001 and IRR 2.3, 95% CI 1.1–4.9, *p* = 0.0247, respectively), (Fig. 2B). In contrast, patients with a low PRODIGY score did not have a significantly different incidence rate ratio for OIRD episodes between when receiving RA and SO (IRR 0.5, 95% CI 0.2–1.0, *p* = 0.0594), (Fig. 2B). Similar results were obtained using a GEE model to determine the IRR for OIRD episodes in the 88 patients who received intermittent SO (Additional File 2).

### Timing of OIRD episodes

The timing of when OIRD episodes occurred in relation to the start of SO was assessed. Sixty-three percent of patients who started continuous monitoring on SO experienced ≥ 1 OIRD episode (*N* = 34/54), compared to 56% of patients who started continuous monitoring on RA (*N* = 19/34), (Table 3). Of the 34 patients who started continuous monitoring on SO, 77% (*N* = 26/34) experienced ≥ 1 OIRD episode before switching to RA. Among the patients who started continuous monitoring on RA, 47% (*N* = 9/19) experienced ≥ 1 OIRD episode before SO began, and 53% (*N* = 10/19) experienced ≥ 1 OIRD episode after SO began (Table 3).

**Table 3:** Incidence of OIRD and timing of SO initiation in patients intermittently receiving SO

Supplemental Oxygen Therapy	Percentage of Patients with ≥ 1 Respiratory Depression Episode
Patient started on supplemental oxygen	63.0% (34/54)
OIRD occurred before room air	76.5% (26/34)
OIRD occurred after room air	23.5% (8/34)
Patient started on room air	55.9% (19/34)
OIRD occurred before supplemental oxygen	47.4% (9/19)
OIRD occurred after supplemental oxygen	52.6% (10/19)

*Abbreviations OIRD* Opioid-induced respiratory depression

**Fig. 3:**
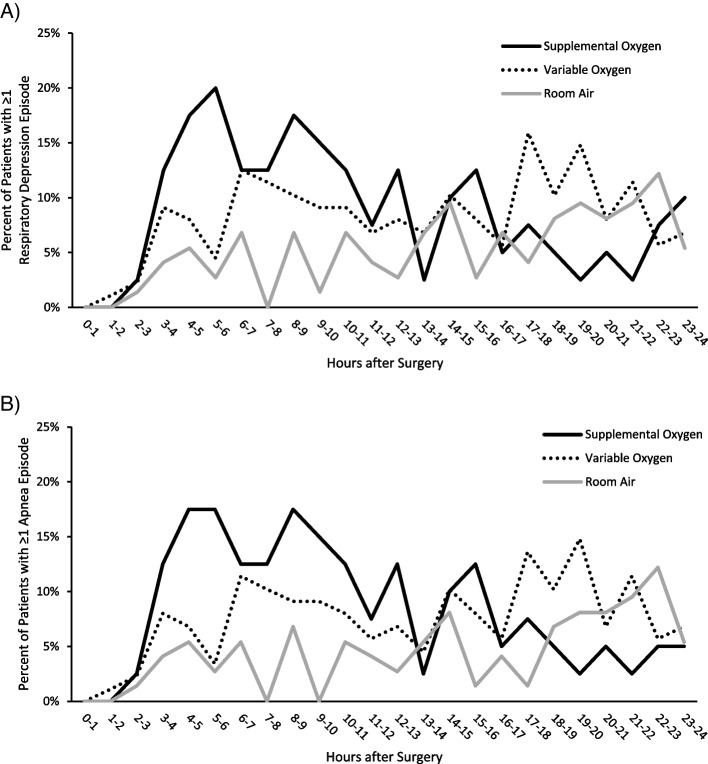
Percentage of patients with **A **≥ 1 respiratory depression episode and **B** ≥ 1 apnea episode as a function of the length of time after surgery. The percent of patients is a population average and does not account for times when continuous monitoring transiently paused

The frequency of patients with ≥ 1 OIRD episode was also assessed as a function of the number of hours after surgery, comparing 40 patients who received SO throughout continuous monitoring, 74 patients always on RA, and 88 patients who received a mix of RA and SO during continuous monitoring. In the first 8-16h after surgery, there was a higher incidence of OIRD among patients who were always receiving SO (Fig. 3A, red line). In contrast, the patients receiving a mix of RA and SO, or who were always on RA, had a lower incidence of OIRD in the first 16h after surgery, and an increased incidence of OIRD 16-24h after surgery (Fig. 3A, blue line). The incidence of apnea events followed a similar trend, with a higher incidence of OIRD among patients always receiving SO in the first 8-16h after surgery, and a higher incidence of OIRD among patients receiving RA or SO 16-24h after surgery (Fig. 3B).

## Discussion

This post-hoc analysis estimated that compared with when patients were on RA, SO was associated with a 2- to threefold increase in the rates of apnea and respiratory rate depression episodes. The design of PRODIGY trial, as well as the lack of strict protocolized criteria for the initiation of SO, do not allow for any safe conclusions regarding the nature of the association between SO and respiratory depression. Even though we cannot exclude a potential role for SO in promoting respiratory depression, the most parsimonious explanation for the observed association is that patients who were at higher risk for respiratory depression were also more likely to receive SO. According to this explanation, the decision of healthcare providers for SO treatment is primarily based on diagnoses and patient-related factors that are widely perceived to heighten the risk for postoperative respiratory depression. However, this is not supported by our findings, since, as shown in Table 1, the most important of these OIRD risk markers, as well as the use of postoperative opioids and sedatives, did not differ among patients on RA, and intermittent or continuous SO. Furthermore, the fraction of patients with a high risk for postoperative respiratory depression, as estimated by the PRODIGY score, did not differ among the groups. The PRODIGY score was devised retrospectively from data obtained via continuous oximetry and capnography monitoring of this same patient cohort. While the healthcare providers who initiated SO were blinded to these data and based their decision on generally accepted patient risk profiles for OIRD, we cannot exclude the possibility that their SO (and/or other) treatment decisions, might have influenced the development of PRODIGY score.

Most observational studies that have examined the use of SO in postoperative patients have focused on its efficacy in preventing or treating hypoxemia and provided variable results, with some supporting a beneficial effect [16–18] and others underlining the ineffectiveness of SO to abolish episodic arterial desaturations that are mainly associated with sleep- or sedation-induced ventilatory disturbances [4, 10, 19]. The use of continuous pulse oximetry monitoring, on the other hand, in contrast to the intermittent SpO_2_ spot-checks [2], has been shown to increase the detection of hypoxemia, and therefore the associated use of SO and/ or naloxone, compared with a group of patients without pulse oximetry monitoring [20]. This is in support of the evidence that the use of continuous pulse oximetry as a sole respiratory monitor in patients on SO might mask the development of hypoventilation [12, 13, 21], potentially precipitating a critical OIRD event in an otherwise well-oxygenated patient.

We postulate that in our cohort, although healthcare providers might have initiated SO based on the criteria discussed above, SO maintenance and intermittent monitoring of oxygenation via pulse oximetry spot-checks, may have precipitated apnea and respiratory depression by concealing hypoventilation in well-saturated patients. Importantly, the majority of the overall 46% incidence of OIRD was determined by capnography, and only a small portion (8%) of it constituted arterial desaturation episodes (i.e., SpO_2_ ≤ 85% for ≥ 3 min), reflecting the fact that a large fraction (62%) of the PRODIGY patients received SO during the monitoring period. Although we cannot exclude a physiological effect of SO on the development of OIRD, our findings support the hypothesis that patients at high risk for OIRD, or those with borderline oxygenation, might had been started on SO, which in turn could have masked a state of escalating hypoventilation, eventually leading to OIRD.

Decreased wakefulness is an important contributory characteristic of pharmacologically induced respiratory depression in postoperative patients [22]. Opioids and residual anesthetics, both directly and via reducing the cortical input to central respiratory control, increase ventilatory instability, promoting the recurrence of apneas/hypopnea episodes that may lead to hypoxemia, even in the presence of SO [19, 23, 24]. Furthermore, both hypoxemia [25] and the transition between sleep and wakefulness, have been shown to exacerbate ventilatory instability, whereas hyperoxia during sleep was found to promote stable breathing [26–28]. In our cohort, patients who were treated with SO throughout the monitoring period showed a peak OIRD rate in the early evening hours (8-16h after surgery), whereas those treated with a mix of RA and SO, or on RA only, experienced a higher incidence of OIRD between 16 and 24h after surgery (Fig. 3). This is an interesting finding, because, according to a previous analysis of the same patient cohort, the rate of *initial* OIRD episodes peaked in the afternoon to early evening hours while the peak rate of *all* OIRD episodes occurred in early morning [15], and might indicate that applying (and maintaining) SO early in the postoperative period may be associated with better stabilization of breathing later during the night and early morning hours when ventilatory instability and higher number of recurring arterial desaturation events have been associated with increased risk of OIRD [29]. In contrast, the application of SO on an “if needed” basis (i.e., mix of SO and RA) on patients who have already developed signs of OIRD and/or hypoxemia, does not seem to affect the natural course of postoperative respiratory depression.

### Limitations

The findings of this post-hoc analysis need to be seen under the light of existing limitations. The use of SO in our cohort was neither randomized, nor dictated by any protocolized criteria, but was determined by local hospital policies and healthcare providers’ judgement. Supplemental O_2_ is not a baseline variable, but it is a time-varying variable, and could be continuous during the entire monitoring period or discontinued once or several times. In this setting, OIRD episodes occurring on SO could be subsequent to episodes occurring on RA. The incidence and frequency of respiratory rate and SpO_2_ episodes were very low and statistical models based on these values could be affected by sample size.

## Conclusions

Although in general oxygen supplementation improves oxygenation in postoperative patients, its presence does not prevent pharmacologically or opioid-induced respiratory depression. Clinicians should practice caution when providing oxygen therapy in that setting and ensure that patients are monitored sufficiently to confirm proper ventilation and intervene before patient deterioration. This post-hoc analysis does not provide a definite answer to the examined relationship between supplemental oxygen therapy and postoperative monitoring of respiration and should be seen as an evidence source for generating future hypotheses.

### Supplementary Information

**Additional file 1.** PRODIGY score. Patient characteristics are assigned a point value, the sum of which determines the patient PRODIGY score (<8 points = low risk; ≥8 and <15 points = intermediate risk; ≥15 points = high risk for opioid-induced respiratory depression).

**Additional file 2. **Generalized estimating equation model for A) the incidence rate ratio of respiratory depression episodes in patients during supplemental oxygen, compared with when on room air, and B) the incidence rate ratio of respiratory depression episodes in patients on intermittent SO (*N*=88).

## Data Availability

The data that support the findings of this study were made available from Medtronic. Restrictions apply to the availability of these data, which are not publicly available. Data are however available from the corresponding author (Anthony Doufas, agdoufas@stanford.edu) upon reasonable request and with permission of Medtronic.

## Abbreviations

ASA: American Society of Anesthesiologists
CI: Confidence interval
CNS: Central nervous system
GEE: Generalized Estimating Equation
IRR: Incidence rate ratio
O_2_: Oxygen
OIRD: Opioid-induced respiratory depression
OSA: Obstructive sleep apnea
PRODIGY: Prediction of Opioid-induced respiratory Depression In patients monitored by capnoGraphY
RA: Room air
SD: Standard deviation
SO: Supplemental oxygen
SpO_2_: Arterial blood oxygen saturation
STROBE: Strengthening the Reporting of Observational studies in Epidemiology guideline
