# Supplementary material for: Incidence of postoperative opioid-induced respiratory depression episodes in patients on room air or supplemental oxygen: a post-hoc analysis of the PRODIGY trial
Source: BMC Anesthesiol. 2023 Oct 4;23:332. doi: 10.1186/s12871-023-02291-x (PMC10548743; doi:10.1186/s12871-023-02291-x)
Supplement: Supplementary file 1 — Additional file 1. PRODIGY score. Patient characteristics are assigned a point value, the sum of which determines the patient PRODIGY score (<8 points = low risk; ≥8 and <15 points = intermediate risk; ≥15 points = high risk for opioid-induced respiratory depression). [file 12871_2023_2291_MOESM1_ESM.docx]

**Additional Files**

**Additional File 1**. PRODIGY score. Patient characteristics are assigned a point value, the sum of which determines the patient PRODIGY score (<8 points = low risk; ≥8 and <15 points = intermediate risk; ≥15 points = high risk for opioid-induced respiratory depression).

| **Patient Characteristic** | **PRODIGY Points** |
| --- | --- |
| Age |  |
| ≥80 | 16 |
| ≥70 - <80 | 12 |
| ≥60 - <70 | 8 |
| <60 | 0 |
| Male sex | 8 |
| Chronic heart failure | 7 |
| Sleep disordered breathing | 5 |
| Opioid naivety | 3 |
| **Sum** | **PRODIGY score** |

**Additional File 2**. Generalized estimating equation model for A) the incidence rate ratio of respiratory depression episodes in patients during supplemental oxygen, compared with when on room air, and B) the incidence rate ratio of respiratory depression episodes in patients on intermittent SO (N=88).

A)

B)
