# Supplementary material for: Incidence of postoperative opioid-induced respiratory depression episodes in patients on room air or supplemental oxygen: a post-hoc analysis of the PRODIGY trial
Source: BMC Anesthesiol. 2023 Oct 4;23:332. doi: 10.1186/s12871-023-02291-x (PMC10548743; doi:10.1186/s12871-023-02291-x)
Supplement: Supplementary file 2 — Additional file 2. Generalized estimating equation model for A) the incidence rate ratio of respiratory depression episodes in patients during supplemental oxygen, compared with when on room air, and B) the incidence rate ratio of respiratory depression episodes in patients on intermittent SO (N=88). [file 12871_2023_2291_MOESM2_ESM.docx]

**Additional File 2**. Generalized estimating equation model for A) the incidence rate ratio of respiratory depression episodes in patients during supplemental oxygen, compared with when on room air, and B) the incidence rate ratio of respiratory depression episodes in patients on intermittent SO (N=88).

A)

B)
